# Multipath Estimation in Urban Environments from Joint GNSS Receivers and LiDAR Sensors

**DOI:** 10.3390/s121114592

**Published:** 2012-10-30

**Authors:** Khurram Ali, Xin Chen, Fabio Dovis, David De Castro, Antonio J. Fernández

**Affiliations:** 1 Department of Electronics and Telecommunications (DET), Politecnico di Torino, Corso Duca degli Abruzzi, 24-10129 Turin, Italy; E-Mail: fabio.dovis@polito.it; 2 Shanghai Advanced Research Institute, Chinese Academy of Sciences, 3rd floor, 1st Building, Haike Road 99, Pudong, Shanghai 201203, China; E-Mail: chenx@sari.ac.cn; 3 DEIMOS Space S.L.U., Ronda de Poniente, 19-28760 Tres Cantos, Madrid, Spain; E-Mails: david.decastro@deimos-space.com (D.D.C.); antonio.fernandez@deimos-space.com (A.J.F.)

**Keywords:** global navigation satellite system, light detection and ranging, multipath

## Abstract

In this paper, multipath error on Global Navigation Satellite System (GNSS) signals in urban environments is characterized with the help of Light Detection and Ranging (LiDAR) measurements. For this purpose, LiDAR equipment and Global Positioning System (GPS) receiver implementing a multipath estimating architecture were used to collect data in an urban environment. This paper demonstrates how GPS and LiDAR measurements can be jointly used to model the environment and obtain robust receivers. Multipath amplitude and delay are estimated by means of LiDAR feature extraction and multipath mitigation architecture. The results show the feasibility of integrating the information provided by LiDAR sensors and GNSS receivers for multipath mitigation.

## 1. Introduction

In Global Navigation Satellite System (GNSS), the user computes its position by means of measured distances between the receiver and a set of in-view satellites. These distances are calculated estimating the propagation time of signals transmitted by each satellite. For such a reason GNSS receivers are only interested in estimating the delays of signals in line-of-sight (LOS) carrying the information on the relative distance. In some environments, reflected versions of the LOS (multipath) may exist due to obstacles surrounding the receiver [1]. When such reflected rays have a delay smaller than one chip duration, they are not mitigated by the shift-orthogonality properties of the codes used for the ranging, and they may cause a bias in the delay and carrier-phase estimations. As a result, the positioning accuracy of GNSS receiver is badly affected.

The problem of multipath mitigation has been extensively tackled in literature [2], since the early years of GNSS. Several techniques have been studied and developed along the years, in order to mitigate the effects in the pseudorange computation [3]. To further improve the robustness to multipath, the recent trend in multipath mitigation is the use of more complex receiver architectures able to estimate the multipath features, thus enabling a mitigation based on a wipe-off of the spurious components. A different approach in order to increase the robustness of the receiver is the coupling of the GNSS receiver with sensors of different nature, with complementary features with respect to the GNSS receiver. Such techniques include the integration of GNSS signal with other information sources and sensors, e.g., inertial navigation system (INS) [4].

In this paper, we present a preliminary work performed, targeting as a final objective the assessment of the integration of the GNSS receiver with a Light Detection and Ranging (LiDAR) system. LiDAR is an optical remote sensing technology that can measure the distance to a target, or other properties of the target, by illuminating the target with light, often using pulses from a laser. LiDAR can be used with a wide range of targets including metallic and non-metallic objects like buildings in urban canyons. A narrow laser beam can be used to map physical features with very high resolution, which is a key point in modeling close reflections.

A LiDAR can be used to obtain digital surface models of the environment, thus using it as an external source of information on the expected reflections impinging the receiver antenna [5]. The objective of this work is (1) to investigate the feasibility of using the LiDAR measurements for the estimation of the GNSS multipath reaching the receiver, thus to cross-check in an urban environment and (2) to validate the possibility to have a run-time estimation of the environment and get aiding information by it. Modeling of the environment surrounding the receiver, taking into account the specific features of GNSS measurements, is becoming of growing importance in order to define the reliability of the position obtained. Classical integrity determination procedure are in fact useless in urban environment where the impact of the multipath ask for different definitions of the protection levels [6,7].

The work presented in this paper is performed by means of real GPS signals and LiDAR measurements recorded during the data collection campaign in an urban environment. The raw GPS data were processed in order to characterize the multipath error using a GNSS fully-software receiver implementing the Coupled Amplitude Delay Lock Loop (CADLL) architecture [8–10], able to estimate the multipath features. Results obtained from this test campaign show the feasibility of the developed approach for multipath error characterization.

The paper is organized as follows: Section 2 briefly summarizes the CADLL architecture, while Section 3 describes the methodology for the data collection and the environment characterization. Section 4 presents an example of multipath estimation and eventually Section 5 draws some conclusions.

## 2. The CADLL Architecture for Multipath Estimation and Mitigation

The CADLL architecture was introduced in [8] and [10] as a multiple DLL structure able to estimate amplitude, delay and phase of a number of relevant reflections of the GNSS LOS signal, in order to cancel them from the incoming signal, thus improving the positioning performance.

**Figure 1.**
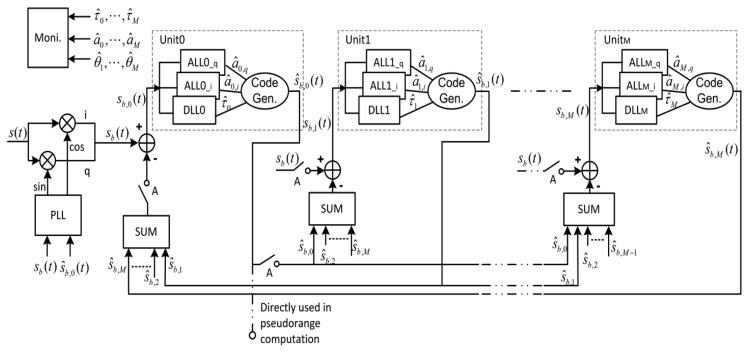
CADLL block diagram.

As shown in Figure 1, CADLL is composed of several tracking units, each tracking a component signal. After a transient time, the estimated multipath signals are subtracted from the total input signal to reduce the error caused by multipath in LOS signal. Inside each tracking unit, a normal code DLL with wide or narrow spacing is used to track the code phase and two Amplitude Lock Loops (ALL) are used to track the amplitudes of the I and Q components of the signal. The ALL is composed of an estimator, a loop filter and an integrator.

The working procedure of CADLL is made of different steps. It first uses a conventional tracking loop to lock onto the incoming signal, getting a rough estimation of the LOS code phase; then it activates two units in order to try to track a multipath signal. If it fails, this means there is no multipath in the incoming signal; if it succeeds, it will continue to try to insert a new unit into this feedback loop to look for a new multipath component.

**Figure 2.**
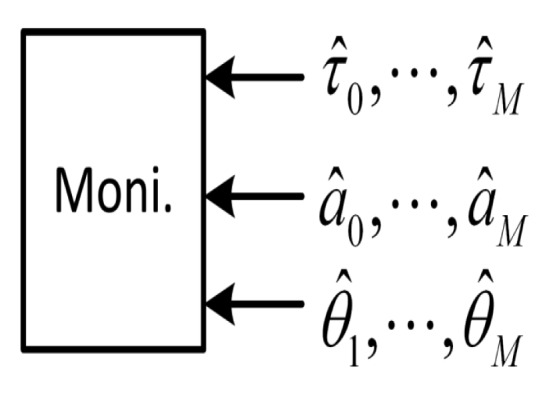
CADLL monitoring unit.

The monitor block (see Figure 2) is governing the process of searching a new multipath component by checking the tracking results of each unit. If it is considered that there is no new multipath component, the trial unit will be shut down by the monitor block. The monitor block checks in particular two parameters: the time difference between the delays estimated by two consecutive units,

(1) $$E\left({\hat{\tau }}_{n-1}-{\hat{\tau }}_{n}\right)<\delta t_{th2}$$

and the amplitude of each tracked component,

(2) $$E\left({\hat{a}}_{n}\right)<{\hat{a}}_{\text{thre}}$$

In order to warn if the amplitude is negligible and if two very close components are tracked, thus resulting in the cancelation of the useful LOS during the feedback phase. The process will not stop until there is no new multipath found or the number of enabled units reaches the maximum number *M_M_*, which is pre-defined according to available resources.

Following this specially designed working procedure, CADLL actually has the ability of estimating the number of multipaths and adjusting its structure to match it. The detailed description of the CADLL working procedure can be found in [8] where the mathematical derivation and performance evaluation are presented.

## 3. Data Collection and Methodology

**Figure 3.**
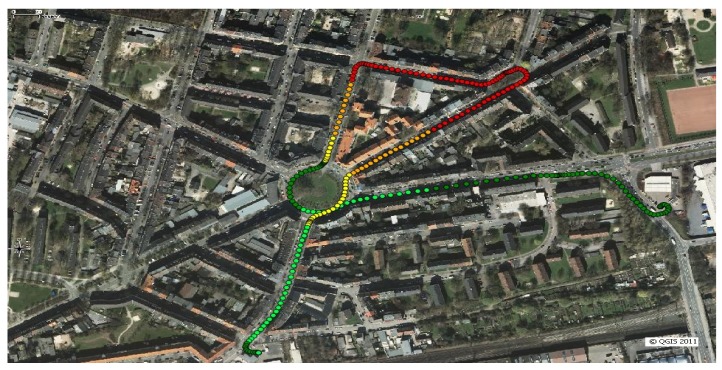
Field data collection path.

A field data collection campaign was carried out at Dortmund, Germany. The test scenario was selected so that the test vehicle followed a pre-defined trajectory along a 1.7 km long closed path. As previously remarked, the objective of the work is to assess the presence of obstacles generating multipath reflections using both GPS and LiDAR observables. The path shown in Figure 3 covers an urban environment, with two lane streets and 4-storey buildings. The buildings fronts are mainly planes parallel with respect to the axis of the streets. Some lampposts and trees are also present on the side of the streets. Figure 3 shows the reconstructed trajectory based on the GPS measurement performed by a professional receiver. Green dots indicate good GPS visibility and measurements accuracy, while red dots correspond to areas in which GPS quality is degraded due to poor visibility and multipath.

**Figure 4.**
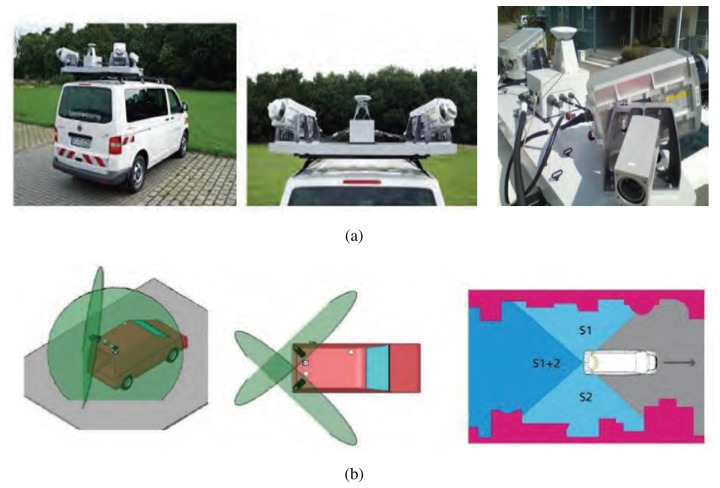
(**a**) Optech Lynx LiDAR sensors equipment; (**b**) Optimum orientation of 2 LiDAR sensors.

Figure 4(a) shows the LiDAR sensors equipment that was used in the test campaign while Figure 4(b) shows the optimum orientation of 2 LiDAR sensors. More details on the equipment used to carry out the test can be found in [11–13].

**Figure 5.**
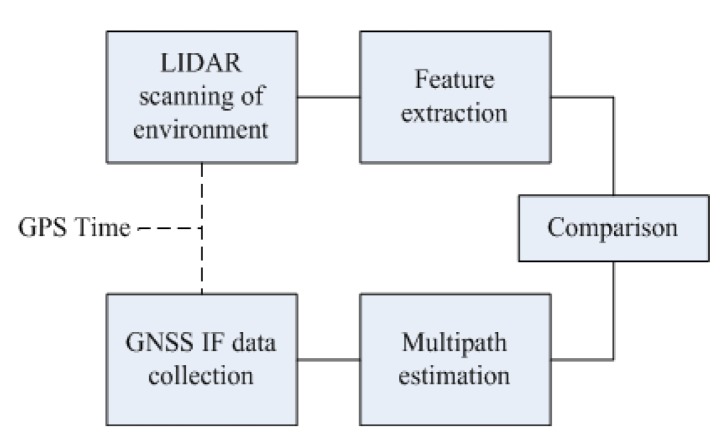
Block diagram of the process.

The block diagram shown in Figure 5 describes the methodology to perform the test. The LiDAR measurements and GPS raw measurements (in-phase and quadrature samples of the received signal at intermediate frequency) from the output of antenna front end are recorded at the same GPS time.

The LiDAR measurements are post-processed in order to extract the information regarding planes of buildings fronts. The raw output of the LiDAR is the so-called cloud of points represented by azimuth and elevation with respect to the phase center of the LiDAR, of the source of reflection. By processing the cloud of points, information like shape, orientation of planes and size of objects can be obtained. For this analysis, planes have been reconstructed by the cloud of points and their distances from the receiver are the features extracted from the LiDAR cloud of points.

**Figure 6.**
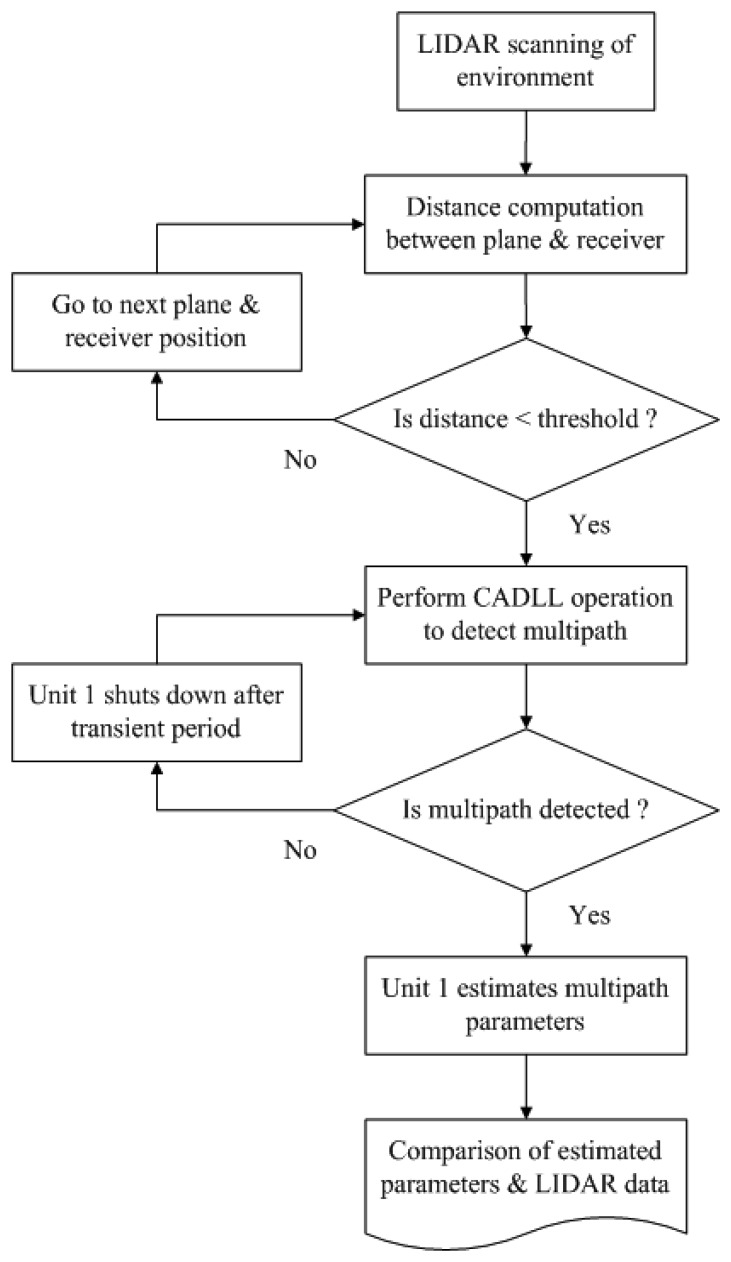
Data processing flow chart.

GPS raw data are post-processed using CADLL to estimate the multipath present in the received signal. CADLL estimates the delays and amplitudes of multipath, which are further translated into the distances of the reflector generator with respect to the center of phase of the GNSS antenna. The information from both the LiDAR feature extraction and GNSS multipath estimation is then compared to check the correlation between the two measurements (see Figure 6).

It has to be remarked that GPS and LiDAR observables are collected in a synchronized manner, by using a common reference clock. Furthermore, the LiDAR+CADLL system is tuned in order to exclude delays larger than one chip duration (and that would not affect the pseudorange measurement), in order to reduce the computational time of the CADLL. In fact, it has to be remarked that in principle, CADLL is able to track components with larger delay, as proved in [14,15].

Only if the LiDAR distances fall within the threshold (300 m), the CADLL operation starts, initializing the two starting units. Unit 0 tracks the direct signal while unit 1 tracks multipath signal if there is any.

## 4. Results and Discussion

**Figure 7.**
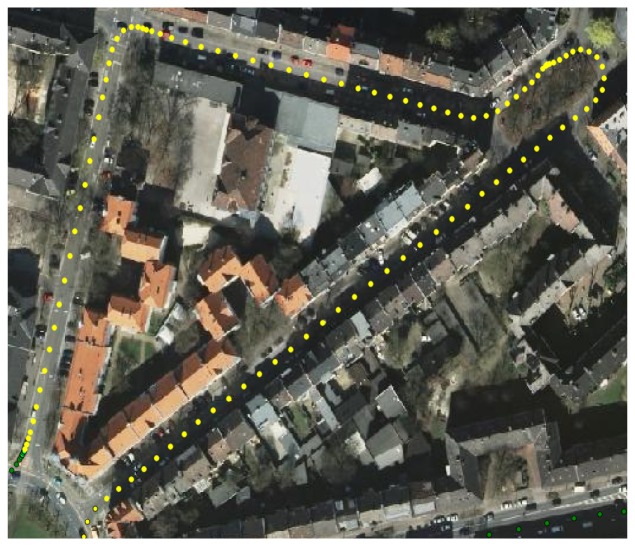
Path segment with close buildings and trees.

The environment chosen is representative of various conditions that may appear in urban and suburban scenarios. In particular, path segments where signal attenuation and multipath were observed are highlighted in Figure 7.

**Figure 8.**
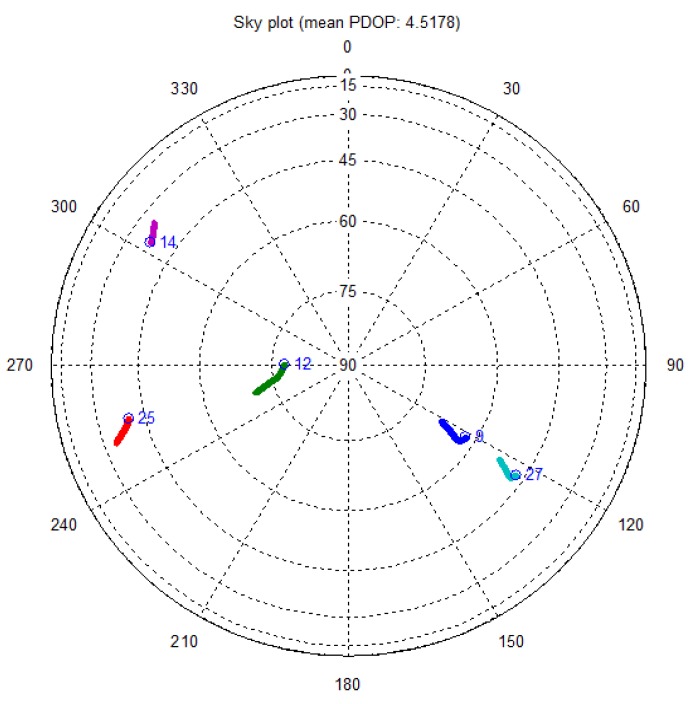
Sky plot for the acquired satellites.

In total, five satellites are acquired during the test campaign. The sky plot for the acquired satellites is shown in Figure 8. Satellite vehicle (SV) 9, SV 12 and SV 27 have good and similar elevation while the others are evenly distributed but mostly at low elevation. In fact, for SV 14 and SV 25, there is strong signal attenuation all along the path at most of the time.

**Figure 9.**
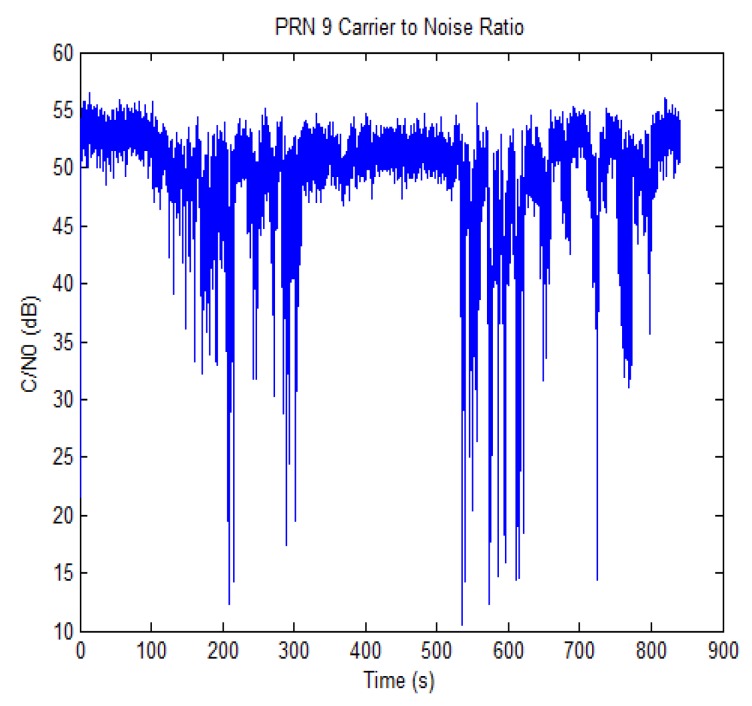
Estimated *C/N*_0_ behavior for SV 9.

The carrier to noise ratio (*C/N*_0_) for one of the satellites in view is shown in Figure 9 (SV 9). The trend shows a good availability of *C/N*_0_ for some time intervals, while for others either signal blockage or strong attenuation is experienced, as it can be noted for the time intervals from 100 to 300 s and from 520 to 840 s. The *C/N*_0_ drop is common to all the satellites during such intervals, and this reveals that the attenuation is due to obstacles close to the receiving antenna, such as the presence of foliage of the trees on the side of the street [16].

In order to confirm the previous inference, the CADLL was used to analyze the GNSS signals in such intervals. Despite the drop in the *C/N*_0_, there were no multiple reflections detected in such time intervals of data collection. It has to be remarked that CADLL considers only multipath that is relevant in terms of distortion of the tracking curve (see Equations (1) and (2)). When only unit 0 is activated, if multipath is present, its effect is irrelevant for the GNSS receiver in terms of bias, even if they may have increased the noise floor and thus reduced the *C/N*_0_. The final conclusion is then that, in such time intervals, the *C/N*_0_ drop must be due to diffused multipaths and scattering (which could actually be generated by the foliage or by the building edges), but no specular multipath is present.

However, processing the full set of data collected by means of the CADLL, segments of the path where specular multipath is present are found. Due to the dynamic data collection, the propagation conditions change quickly and the specular multipath is detected for short durations. As an example, multipath is detected on SV 9 at 475 s, for a duration of about 12 s.

**Figure 10.**
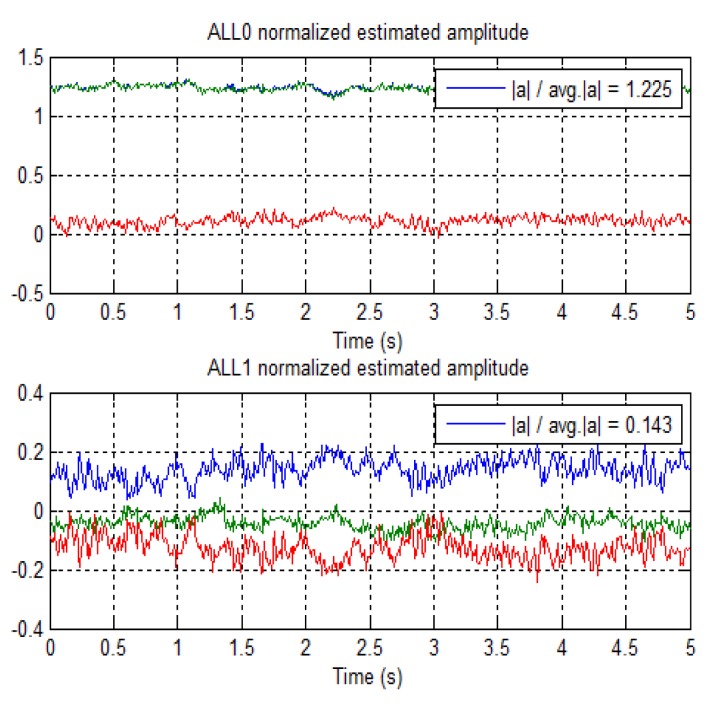
Estimated amplitudes by unit 0 (top) and unit 1 (bottom) in the time interval from 475 to 480 s.

Figure 10 shows the trend of the normalized amplitude for the detected direct and multipath rays starting at time epoch 475 s. ALL0 and ALL1 are estimating the amplitudes of the LOS and multipath signals respectively. It is noted that the multipath amplitude is quite low with respect to the LOS amplitude, showing that the multipath is a very weak. In the figure, green and red lines are the respective I and Q components of the complex signal. For this case, the average estimated power ratio between the reflected ray and the LOS is −19 dB.

**Figure 11.**
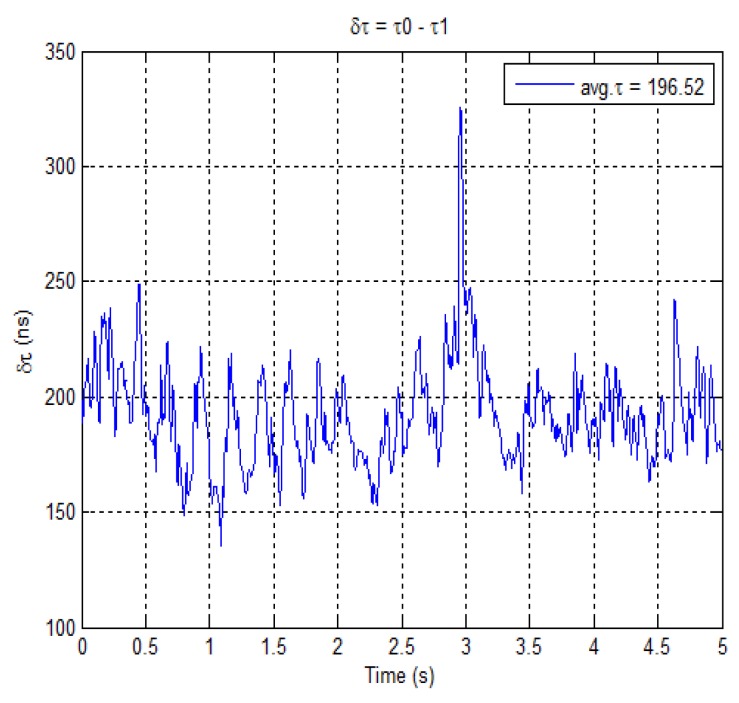
Estimated delay difference between unit 0 and unit 1.

Figure 11 depicts the delay trend between the direct and the multipath signal. The average delay between the two signals is estimated to be about 196.5 ns. It is important to note that for this duration, the multipath is close to the direct signal. Also the trend is fairly steady and without significant changes, thus making likely that it is reflected from the same source during the whole time interval. The estimated delay in this case corresponds to a difference in traveling path of about 58 m. This estimation is in good agreement with the data collection performed by the LiDAR. In fact, by analyzing the distribution of planes as obtained through processing the cloud of points, it can be found that there are planes at distances in the range of 50–60 m.

**Figure 12.**
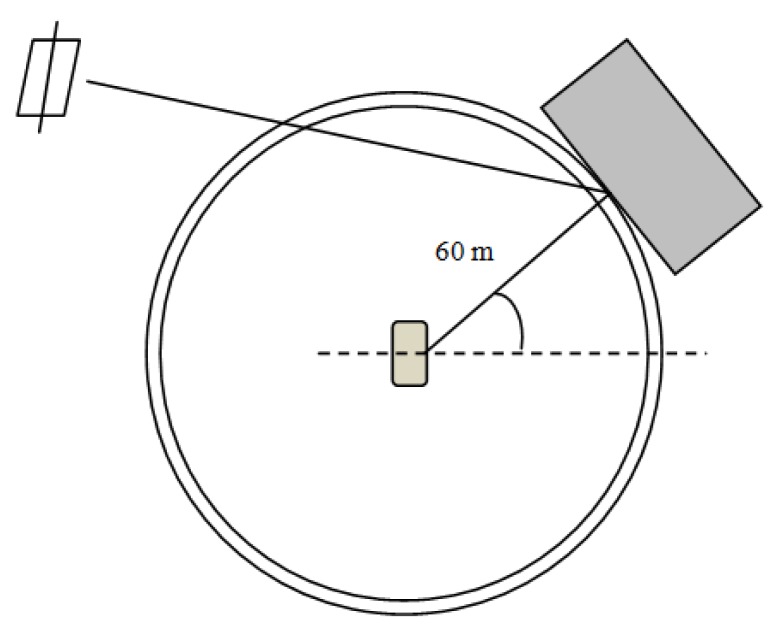
Description of multipath estimation scenario.

The multipath estimation scenario is depicted in Figure 12 where it is shown how the distances, between the receiver and the object causing multipath, both from GNSS and LiDAR are related. This fact validates the outputs of the CADLL processing and this example shows how the output of the LiDAR might be used to estimate the possibility of having multipath biases in the measurements.

## 5. Conclusions and Future Work

In this paper, a preliminary work on the feasibility of using LiDAR measurements for estimating the likelihood of multipath presence is presented. From the estimation of planes obtained by the LiDAR cloud of points and the knowledge of the satellite positions, the multipath on GNSS might be estimated. However, this would only justify the possibility of the multipath, the presence of which is dependent also on the material and the reflection properties of the obstacles. However, the example presented in this paper also shows that this kind of detection would not have been possible by just observing the *C/N*_0_; in fact the signal drop is masked by the variance of the measurement of the signal-to-noise ratio in an urban environment. As a side results of this work, it has also been shown that significant drops in the *C/N*_0_ in urban environment are more often due to scattered multipath and that such phenomenon can be identified since it is common to all the satellites in view.

As further work, deeper integration of the information obtained by the LiDAR and the GNSS receiver is foreseen, with the twofold objective of designing an “environment analyzer” able to actually report multipath that is relevant for GNSS receivers and applications, by merging the information derived by the two sources.
